# Recent Developments in Vascular Imaging Techniques in Tissue Engineering and Regenerative Medicine

**DOI:** 10.1155/2015/783983

**Published:** 2015-03-02

**Authors:** Paul Kumar Upputuri, Kathyayini Sivasubramanian, Chong Seow Khoon Mark, Manojit Pramanik

**Affiliations:** Nanyang Technological University, School of Chemical and Biomedical Engineering, 70 Nanyang Drive, Singapore 637457

## Abstract

Adequate vascularisation is key in determining the clinical outcome of stem cells and engineered tissue in regenerative medicine. Numerous imaging modalities have been developed and used for the visualization of vascularisation in tissue engineering. In this review, we briefly discuss the very recent advances aiming at high performance imaging of vasculature. We classify the vascular imaging modalities into three major groups: nonoptical methods (X-ray, magnetic resonance, ultrasound, and positron emission imaging), optical methods (optical coherence, fluorescence, multiphoton, and laser speckle imaging), and hybrid methods (photoacoustic imaging). We then summarize the strengths and challenges of these methods for preclinical and clinical applications.

## 1. Introduction

Cellular function and viability are highly dependent on the effective diffusive exchange of nutrients and metabolic waste through tissue.* In vivo*, most cells are found within 200 micrometres away from the nearest capillary to achieve this and vasculopathic conditions, such as seen in patients suffering from diabetes and cardiovascular diseases, result in poor tissue perfusion, eventually leading to ischemia and necrosis. Aside from diseases, ischemia is also seen in transplanted tissues, leading to compromised graft viability and “take.” This is exacerbated in tissue engineered products, which are typically avascular, and remains a major bottleneck in the clinical translation of engineered tissues from bench to bedside. Thus, there is an immense need for revascularisation strategies to stimulate regeneration of vascular networks and reverse ischaemia [1]. Specifically, in tissue engineering, various research efforts are directed towards accelerating vascularisation following implantation [2].

These strategies typically involve the use of biological and/or pharmaceutical agents to elicit angiogenesis and vasculogenesis. Angiogenesis refers to the sprouting of capillaries and vasculature from existing blood vessels and is orchestrated by various growth factors and cytokines [3]. The process is initiated by angiogenic factors (such as vascular endothelial growth factor; VEGF) binding to receptors on endothelial cells in existing blood vessels, leading to activation of mitogenic pathways and establishment of a chemotactic gradient. Consequently, endothelial proliferation and migration result in capillaries sprouting from the parent vessel towards the site of interest. In contrast, vasculogenesis refers to the de novo formation of blood vessels formed by endothelial or precursor cells. The process is initiated by the migration and aggregation of progenitor cell populations in a tissue space, spontaneously forming new vasculature. Although the two processes may work in tandem to give rise to the same desired clinical outcome, it may be useful to distinguish them at this stage. For example, revascularisation by endothelial progenitor cells arises from angiogenesis and vasculogenesis [4], while mesenchymal stem cells exert primarily angiogenic effects [5].

Despite significant advances in our understanding of the process of tissue vascularisation, further progress is currently hampered by the lack of tools to visualise and quantify these observations* in vivo*. Many imaging modalities have emerged in recent years to address this need and will be discussed in the subsequent section of this review paper.

## 2. Visualising the Process of Vascularisation* In Vivo*

In traditional methods of animal experiments, Doppler perfusion imaging is often performed on downstream tissue, as an indirect measure of vascularisation. However, this neither does allow visualisation of the blood vessels nor does provide any information on the status of emergent vasculature. Thus, tissue vascularisation is hitherto quantifiable only through terminal experiments, involving histological sections or whole mount imaging of the harvested tissue [16]. Thus, longitudinal assays are not possible, severely limiting experimental designs and reliability of the findings. Similarly, in the clinical setting, noninvasive imaging techniques to monitor vascularisation and reperfusion as measures of clinical outcome is essential [17]. It is thus imperative to develop imaging modalities capable of visualising vascular networks* in vivo*. To this end, the ideal imaging modality should be able to achieve sufficient tissue penetration and resolution to distinguish sprouting microvasculature. Additionally, vascularisation processes take place on a time scale of days and serial images must be sufficiently robust to allow meaningful quantification. Finally, desirable characteristics include concurrent acquisition of functional data, including tissue perfusion.

### 2.1. Nonoptical Methods

The nonoptical methods widely used for vascular imaging are X-ray/CT, MRI, ultrasound (US), and positron emission tomography (PET). X-ray imaging works based on the attenuation of X-ray inside the body by different tissues. It has been used for many years to examine the large blood vessels. A more advanced technology,* computed tomography* (CT), allows 3D visualisation of the vessels and its surrounding structures. Micro-CT (*μ*CT) [18, 19] is able to provide much higher resolution (~1 *μ*m) imaging, better than ultrasound (~30 *μ*m) and MRI (~100 *μ*m). It allows visualization and quantification of microvasculature with the use of contrast agents (radio opaque and radio dense contrast agents).

**Figure 1:**
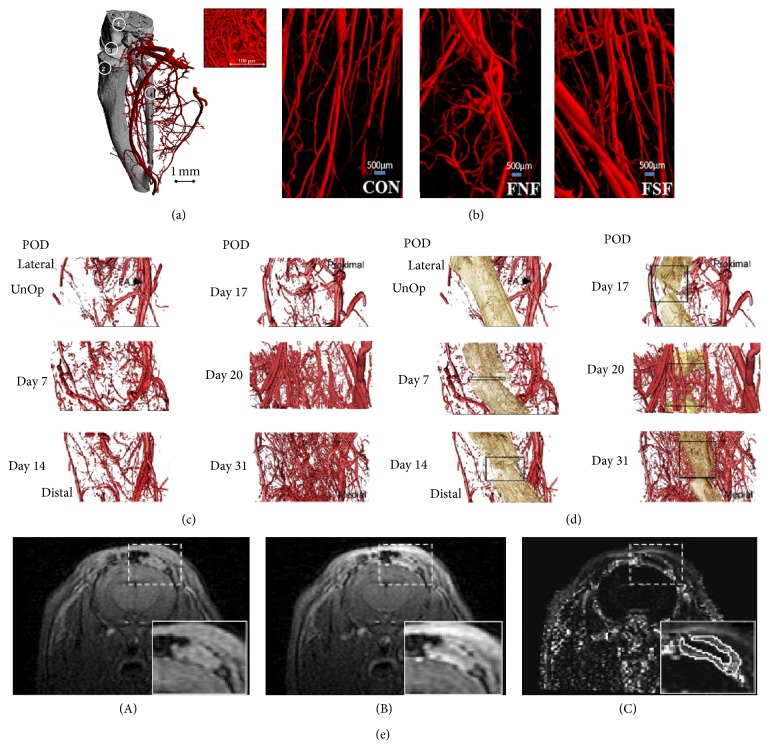
(a) Micro-CT image (at 10 *μ*m resolution) of a vascular corrosion cast of the right lower hind limb (blood vessels in red, bone in grey). Numbers represent anatomical structures: (1) femur; (2) tibia; (3) knee joint; (4) fibula. Inset image is the micro-CT image (at 1.4 *μ*m resolution) of microvasculature. Figure reproduced from [6] with permission. (b) Micro-CT imaging of the 3D vasculature of the soft tissue around the fracture where CON-control group, FNF-fracture with no fixture, and FSF-fracture with surgical fixture. Figure reproduced from [7] with permission. Representative renderings of (c) the vasculature and (d) the vasculature + mineralized tissue across the time-course of distraction osteogenesis (vascular tissues, red and mineralized tissue, tan). The position of the femoral artery (FA) is denoted by an arrow in the renderings of the unoperated controls. Boxes are approximations of the total width of the distraction gap at each time-point. Figure reproduced from [8] with permission. (e) T1-weighted images (A) before and (B) 60 s after Gd-DTPA injection, (C) the corresponding IAUC60 map. A zoomed-in version of the implanted VEGF-impregnated soft tissue construct is shown in inset. Figure reproduced from [9] with permission.

Recently *μ*CT was used for the visualisation of the blood vessels using a densitometric approach and found that the 10 *μ*m medium resolution *μ*CT allowed only the medium and the large blood vessels to be imaged (Figure 1(a)) [6]. The smaller vessels required 1.4 *μ*m ultra high resolution *μ*CT to visualise them in 3D. This study was done in combination with the traditional morphometric analysis, hence providing complete information about the vascular system. Another study (Figure 1(b)) shows that there is more neomicrovasculature in fracture with no fixture (FNF) than the control (Con) group and there is even more neomicrovasculature in the fracture with the surgical fixture group (FSF) indicating that tissue engineering can induce angiogenesis [7]. Another recent study, on the vasculogenesis with respect to the bone growth or repair, showed that the vascular tissue formation occurs in both the muscular and the osseous region and the intensity of the angiogenesis period was concurrent with that of the osteogenesis period [8]. The vessel imaging was done before and after decalcification process. It was observed that the vessel volume and the vessel connectivity density increased between initial and the final measurement points. During the initial stages of osteogenesis, the larger vessels grew more but towards the end it was the smaller vessels which recorded more growth and spreading (Figures 1(c) and 1(d)). In spite of excellent image quality and high resolution, we have to keep in mind that CT uses ionizing radiation and use of exogenous contrast agents, which are not innocuous.

*Magnetic resonance imaging* (MRI) [20–22], on the other hand, works based on the contrast related to the differences in the density of the proton (hydrogen nuclei, abundantly available in water and fat present in the body). It uses no ionizing radiation, no intra-arterial puncture, and safe contrast agents. MRI provides excellent soft tissue contrast helping with tissue segregation and volume quantification. Angiogenesis was measured with MRI with the help of various functional parameters like blood volume, perfusion, permeability, and vasoreactivity. Various contrast agents aid in this process. New vessel formation (angiogenesis) imaging would be based on the changes in the total blood volume which is a direct indication of neoangiogenesis. These were performed with dynamic contrast enhanced MRI (DCE-MRI) [23]. MRI was used to monitor blood oxygenation. Abnormal vascularization was measured with the help of the contrast agent like albumin Gd-DTPA [23]. A study was done to monitor angiogenesis in soft tissue engineered constructs of calvarium for bone tissue engineering with various contrast agents.  Figure 1(e)(A) shows that the vessels are visible even without any contrast agent. With the contrast agent Gd-DTPA and imaging after 60 seconds improved images were obtained and angiogenesis was observed [9]. The MRI could provide high resolution and soft tissue contrast. However, it faces the following challenges and drawbacks: inconveniently long scan time, expensive and nonportable equipment, probable need for strong magnetic fields for better signal-to-noise ratio, movement artifacts, being not suitable for microvascular imaging, and being not ideal for real-time imaging.

### 2.2. Optical Methods

**Figure 2:**
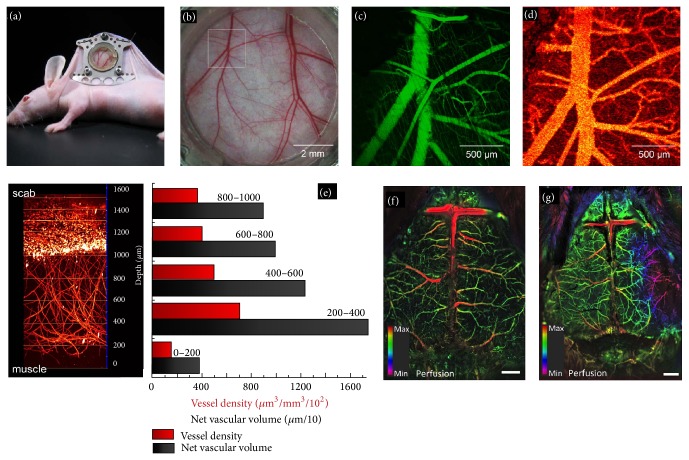
SV-OCT platform for detection of the microvasculature: Photograph (a), white light image (b) of dorsal skinfold chamber. The imaging location is indicated by the box. (c) Maximum intensity projection image of 1PFM, (d) SV-OCT enface projection image of vasculature. Figure reproduced from [10] with permission. Deep-vascular imaging by 2PFM: (e) 3D reconstruction of invading capillaries from successive 1 *μ*m optical sections. Vascular network extends from 0 to approximately 1.1 mm and integrin expressing cells from 1.1 to 1.6 mm. Graph showing the vascular density as a function of section depth. Figure reproduced from [11] with permission. Visualization of hemodynamic changes during an acute ischemic event: (f) images of the cortical vasculature of a control mouse, (g) images of the cortical vasculature following occlusion; the lesion is shown as purple area on the right side. Figure reproduced from [12] with permission.

X-ray/computed tomography (CT), magnetic resonance imaging (MRI), positron emission tomography (PET), and ultrasound imaging (USI) are the most popular clinical methods for macrovascular imaging. Due to the poor spatial resolution or contrast, these are not effective for microvascular imaging. Optical methods have been widely used for microvascular imaging. We further classify the optical methods into two major groups [24, 25]: ballistic (minimally scattered) imaging which can provide better resolution but low penetration depth ~0.5–1.5 mm in tissues, for example, single-photon fluorescence microscopy (1PFM), two-photon fluorescence microscopy (2PFM), orthogonal polarization spectral imaging (OPSI), laser speckle contrast imaging (LSCI), and optical coherence tomography (OCT) and diffusive (multiscattered) imaging which can provide few centimetres imaging depth but poor (one-third of imaging depth) resolution, for example, diffuse optical tomography (DOT).* Optical coherence tomography (OCT)* [26–29] works based on the principle of low-coherence interferometry. It offers label-free, noninvasive, noncontact, and high spatial-temporal resolution cross-sectional imaging in biological tissues. Various OCT vascular imaging techniques have been developed so far [28]. Doppler OCT is an established tool for depth-resolved flow measurements in tissues. However, it is insensitive to flow in the direction normal to the imaging beam. Phase variance (PV) and speckle variance (SV) OCT are the two most recent techniques which can image vasculature structure independent of the vessel orientation and the flow velocity* in vivo* in 3D [10, 28]. However, research efforts are necessary to minimize the artifacts due to vessel shadowing and bulk tissue motion to achieve high resolution imaging in large volume. Figures 2(a)–2(d) show that an* in vivo* imaging of blood flow in vessels (~25 *μ*m in diameter) in a dorsal skinfold window chamber model was successfully demonstrated and compared with intravital fluorescence confocal microscopy [10].

*Single-photon fluorescence microscopy* (1PFM) [30–33], in confocal geometry, is widely used for 3D visualization of the microvasculature. It has high depth resolution, but limited penetration depth (50–200 *μ*m when imaged using visible light) and spatial resolution decreases with depth, and hence it can only be used for thin transparent tissues, but not for large blood vessels such as artery. Recently, 1PFM was incorporated with adoptive optics to correct aberrations, thereby enhancing contrast and sensitivity of vascular imaging.* Two-photon fluorescence microscopy* (2PFM) [11, 34, 35] is a nonlinear imaging modality which is inherently confocal and hence enables 3D visualization of the microvasculature. Its lateral and depth resolutions are 0.3 *μ*m and 0.7 *μ*m, respectively, which are comparable to 1PFM. Conventional 2PFM requires labelling and can usually provide deep tissue imaging at *μ*-resolution over 300 *μ*m depth. Recently, two exciting contributions have been made in 2PFM: one is imaging new vasculature at ~1.6 mm deep within the wounded tissue (Figure 2(e)) [11], and the other is* in vivo* label-free imaging of microvascular morphology and oxygenation [36].

*Orthogonal polarization spectral imaging* (OPSI) [25, 37] in which a special optics creates a virtual light source at a depth of ~1 mm within the tissue. Then the light is absorbed by the haemoglobin (Hb), yielding an image of the illuminated Hb-carrying structures in negative contrast. It enables intrinsic 2D microvascular imaging. Due to the lack of measurement consistency, it is not suitable for chronic changes. However, it can assess quantitative changes in the microvessels and thus allows noninvasive assessment of tissue vascularization. OPSI can only assess total Hb concentration (HbT), but Hb oxygen saturation (SO_2_) is still a challenging issue for both 2PFM and OPSI due to poor SNR [36].

*Laser speckle contrast imaging* (LSCI) [38–40] works based on dynamic scattering of diffusively reflected laser light. It is sensitive to the movement of red blood cells (RBCs) inside vessels and hence is applied for continuous imaging of blood flow dynamics. It allows noncontact, real-time, noninvasive monitoring of skin microvasculature. Recent studies have shown that the reproducibility of LSCI in clinical measurements is much better than laser Doppler flowmetry (LDF) [41], making it an attractive tool for clinical applications. However, ~1 mm depth can be achieved with LDF whereas only ~300 *μ*m with LSCI. Recently a novel optical method which combines LSCI and dynamic fluorescent (DF) imaging was combined with IHS (intensity, hue, and saturation); colour model was proposed for wide-field vascular imaging [12]. The performance of the method was tested to visualize hemodynamic changes by induced occlusion of the middle cerebral artery. Hybrid DFLS images of the cortical vasculature of a control mouse are shown in Figure 2(f) and the following occlusion is shown in Figure 2(g). It clearly demonstrates the area of lesion in the right hemisphere (Figure 2(g)) as purple area.

### 2.3. Hybrid Methods

*Photoacoustic imaging* (PAI), a hybrid imaging method combining high optical absorption contrast with high ultrasound resolution, is highly desirable for vascular imaging and characterization [14, 24, 42–45]. In PAI, the tissue absorbs short laser pulses and generates ultrasonic waves (also known as photoacoustic (PA) waves) which can reveal the physiological information of absorbers such as blood, melanin, and water. PAI is a potential tool for vascular imaging due to the following advantages: (a) it can provide label-free vascular imaging, (b) it has highly scalable spatial resolution (0.1 to 800 *μ*m) and penetration depth (0.1 to 50 mm) higher than 1PFM, 2PFM, OCT, Raman and Coherent Raman microscopy [46], and so forth and hence allows* in vivo* 3D vascular imaging from a superficial capillary (diameter 5 to 10 *μ*m) to an abdominal aorta (diameter 2 to ~3 cm) [25, 47], and (c) it can provide structural and functional information using multiple-wavelength. PAI for visualization and characterization of microvasculature [25, 44] and its applications in tissue engineering have been discussed in detail elsewhere [43, 48]. PAI was combined with several other modalities to provide complementary information for more accurate information. OR-PAM (optical resolution photoacoustic microscopy) is one embodiment of PAI which has been used for angiogenesis study [49, 50], due to its superior resolution to image even capillaries noninvasively.

**Figure 3:**
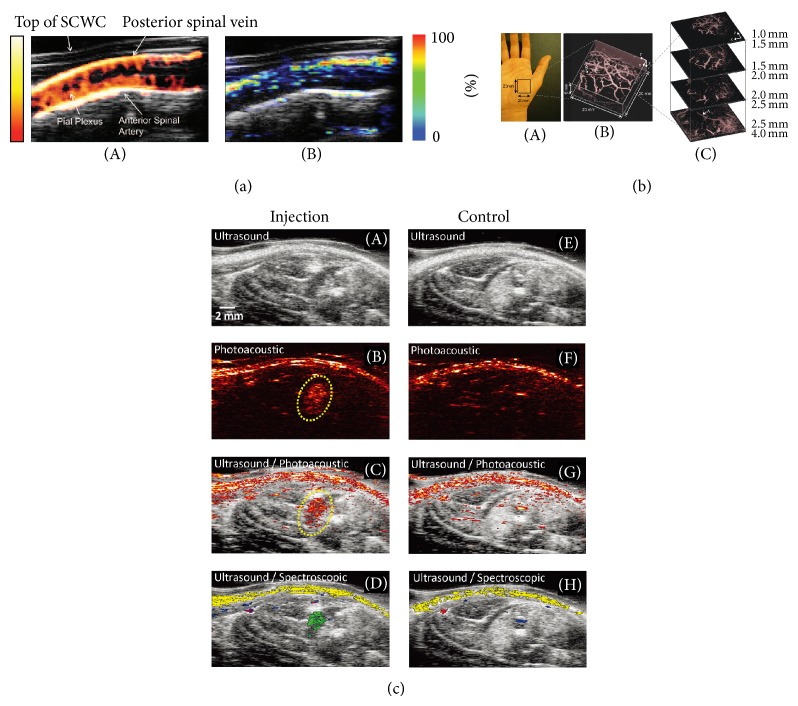
(a) Structural, functional, and oxygenation imaging of the intact spinal cord vasculature in situ: (A) Power Doppler US (color) overlaid on a B-mode structural US (gray-scale) image obtained through the polycarbonate spinal cord window chamber along a longitudinal section of the normal spinal cord* in vivo*. The color bar represents the signal intensity. (B) Corresponding multispectral PA imaging of the same cross section of normal spinal cord permitted in situ measurement of hemoglobin oxygen saturation in the anterior spinal artery and posterior spinal vein. It demonstrated that the cord is well oxygenated. The color bar represents the relative hemoglobin oxygen saturation level. Figure reproduced from [13] with permission. (b) PAI of the vasculature in a human palm* in vivo* with excitation wavelength is 670 nm. (A) Photograph of the imaged region, (B) volume rendered image, and (C) lateral slices at different depths. The arrow A indicates the deepest visible vessel, which is located 4 mm beneath the surface of the skin. Figure reproduced from [14] with permission. (c)* In vivo* monitoring of Au NT labeled MSCs using US/PA imaging: (A–D) i*n vivo* US, PA, US/PA, and US/spectroscopic images of the LGAS in which PEGylated fibrin gel containing Au NT loaded MSCs (1 × 10^5^ cells/mL) was injected. PEGylated fibrin gel location is outlined with yellow dotted circle. Injection depth was about 5 mm under the skin. (E–H) Control at the region of the LGAS of the other hind limb without any injection. Spectral (650–920 nm) analysis of PA signal was able to differentiate between skin (shown in yellow), oxygenated (red) and deoxygenated (blue) blood, and Au NT loaded MSCs (green). The images measure 23 mm laterally and 12.5 mm axially. Figure reproduced from [15] with permission.

Recently, PAI was combined with USI to monitor blood oxygen saturation (BOS) in the scaffolds in tissue engineering [51] and vascular growth in tissue engineered constructs [52]. A multiscale PAI was combined with OCT to investigate neovascularization in 3D porous scaffolds [53].* In vivo* imaging of murine spinal cord and its vasculature was reported using multimodal US/PA imaging technique [13].* In vivo* power Doppler US and PA were used to directly visualize the cord and vascular structures and to measure hemoglobin oxygen saturation through the complete spinal cord, respectively.  Figure 3(a) shows the vascular network of the spinal cord using power Doppler US imaging (A) and SO_2_ measured using multispectral PA imaging (B). Since power Doppler is more sensitive to the detection of small vessels compared to photoacoustics, the data shown in Figure 3(a)(A) displayed an increased number of vascular structures in comparison to Figure 3(a)(B).  Figure 3(b) shows that an* in vivo* PA image of the subcutaneous vasculature in the human palm was obtained using the 38 *μ*m Fabry–Perot sensor [14]. Vessels ~4 mm beneath the skin are clearly visible in Figure 3(b)(C). The US/PA imaging was reported as potential tool for* in vivo* monitoring of mesenchymal stem cells (MSCs) and neovascularization promoted by MSCs [15]. As the MSCs have poor optical absorption, they were labelled with gold nanotracers (Au NTs) and injected intramuscularly in the hind limb of the rat and visualized using US/PA imaging, Figure 3(c)(A–D). The US image shows the structural information of the lower limb, but the location of MSCs cannot be identified. However, the PA and US/PA images clearly show the location of the nanotracer signal from MSCs in the gel outlined in yellow. In order to distinguish the PA signal from the Au NT labelled MSCs from surrounding tissue, spectral analysis was performed, which augments PA imaging. As shown in Figure 3(c)(D), the Au NT labelled MSCs (shown in green) can be clearly distinguished from other tissue constituents, such as oxygenated (red) hemoglobin, deoxygenated (blue) hemoglobin, and skin (yellow), by spectral analysis. A region of the lateral gastrocnemius (LGAS) of the other hind limb without any injection served as a control as shown in Figure 3(c)(E–H).

Finally, we summarize all the vascular imaging modalities reviewed and their key parameters such as imaging contrast, resolution, penetration, and anatomical and physiological parameters that can be imaged in a tabular form as shown in Table 1.

## 3. Discussion

We discussed the recent developments in the vascular imaging methods for tissue engineering applications. CT and MRI are expensive and bulky, but due to its potential for high resolution structural and functional vascular imaging using contrast agents, it is still preferred in clinical applications. The USI is feasible for clinical studies, but its low spatial resolution (~30 *μ*m) and lack of functional information limit its applications for microvascular imaging. OPSI can only assess total haemoglobin concentration (HbT), but Hb oxygen saturation (SO_2_) is still a challenge for both 2PFM and OPSI due to poor SNR. Both SV- and PV-OCT have unique strengths and weaknesses that make them useful in different preclinical and clinical situations. However, both require improvements to minimize the artifacts due to vessel shadowing and bulk tissue motion to achieve high resolution imaging in large volume. PAT has the potential to be a mainstream technology in microvascular imaging and characterization. However, there are several challenges and immediate tasks to make it even better tool for clinical applications: (a) developing a miniaturized handheld PAI system, (b) although NRI wavelengths allowed deep-vascular imaging, there is a need for exogenous contrast agents or efficient imaging techniques to improve it further, and (c) PA signal is a function of optical absorption coefficient and local fluence. To make it a robust quantitative measurement tool, the local fluence must be compensated accurately.

## Figures and Tables

**Table 1:** Summary of nonoptical, optical, and hybrid imaging modalities for vascular imaging in tissue engineering [18, 21, 25, 28, 54].

	Modality	Imaging contrast	Spatial resolution (*µ*m)	Imaging depth (mm)	Anatomical/physiological parameters
Nonoptical method	X-ray/CT^1^	X-ray absorption	100	Full body	Bone structure, blood vessels imaging (with contrast agent)
	MRI	Tissue relaxation (T1, T2), proton density	25^2^–100	Full body	Soft tissue structure, blood vessels imaging (with contrast agent)
	US	Ultrasound scattering	30	300	Soft tissue structure, blood flow (Doppler ultrasound)
	PET	Radioisotope concentration	1000	Full body	Blood flow

Optical method	OCT	Optical scattering	1–10	1-2	Blood flow, hemoglobin oxygen saturation (SO_2_)^3^
	1PFM	Fluorescence, scattering	1-2	0.2–0.5	Microvascular morphology, blood flow
	2PFM	Fluorescence	1-2	0.5–1.0	Microvascular morphology, blood oxygenation
	OPS	Optical absorption	1–5	0.5–1.0	Total hemoglobin concentration (HbT)
	LSI	Blood flow	10	0.1–0.3	Blood flow

Hybrid method	PAI	Optical absorption	0.1–800	0.1–70	HbT, SO_2_, blood flow, blood vessel structure

^1^micro-CT can provide 1 *µ*m resolution with limited imaging depth.

^2^with very high strength magnetic fields.

^3^It can be measured by integrating OCT with hyperspectral imaging.

## References

[1] Silvestre J.-S., Smadja D. M., Lévy B. I. (2013). Postischemic revascularization: from cellular and molecular mechanisms to clinical applications. *Physiological Reviews*.

[2] Auger F. A., Gibot L., Lacroix D. (2013). The pivotal role of vascularization in tissue engineering. *Annual Review of Biomedical Engineering*.

[3] Yoo S. Y., Kwon S. M. (2013). Angiogenesis and its therapeutic opportunities. *Mediators of Inflammation*.

[4] Liu Y., Teoh S. H., Chong M. S. (2013). Contrasting effects of vasculogenic induction upon biaxial bioreactor stimulation of mesenchymal stem cells and endothelial progenitor cells cocultures in three-dimensional scaffolds under *in vitro* and *in vivo* paradigms for vascularized bone tissue engineering. *Tissue Engineering Part A*.

[5] Kinnaird T., Burnett E. S., Shou M. (2004). Local delivery of marrow-derived stromal cells augments collateral perfusion through paracrine mechanisms. *Circulation*.

[6] Nebuloni L., Kuhn G. A., Vogel J., Mulüler R. (2014). A novel in vivo vascular imaging approach for hierarchical quantification of vasculature using contrast enhanced micro-computed tomography. *PLoS ONE*.

[7] Zhao F., Zhou Z., Yan Y. (2014). Effect of fixation on neovascularization during bone healing. *Medical Engineering & Physics*.

[8] Morgan E. F., Hussein A. I., Al-Awadhi B. A. (2012). Vascular development during distraction osteogenesis proceeds by sequential intramuscular arteriogenesis followed by intraosteal angiogenesis. *Bone*.

[9] Beaumont M., DuVal M. G., Loai Y., Farhat W. A., Sándor G. K., Cheng H.-L. M. (2010). Monitoring angiogenesis in soft-tissue engineered constructs for calvarium bone regeneration: an *in vivo* longitudinal DCE-MRI study. *NMR in Biomedicine*.

[10] Mariampillai A., Standish B. A., Moriyama E. H. (2008). Speckle variance detection of microvasculature using swept-source optical coherence tomography. *Optics Letters*.

[11] Yanez C. O., Morales A. R., Yue X. (2013). Deep vascular imaging in wounds by two-photon fluorescence microscopy. *PloS ONE*.

[12] Kalchenko V., Israeli D., Kuznetsov Y., Harmelin A. (2014). Transcranial optical vascular imaging (TOVI) of cortical hemodynamics in mouse brain. *Scientific Reports*.

[13] Figley S. A., Chen Y., Maeda A. (2013). A spinal cord window chamber model for in vivo longitudinal multimodal optical and acoustic imaging in a murine model. *PLoS ONE*.

[14] Zhang E. Z., Laufer J. G., Pedley R. B., Beard P. C. (2009). In vivo high-resolution 3D photoacoustic imaging of superficial vascular anatomy. *Physics in Medicine and Biology*.

[15] Nam S. Y., Ricles L. M., Suggs L. J., Emelianov S. Y. (2012). In vivo ultrasound and photoacoustic monitoring of mesenchymal stem cells labeled with gold nanotracers. *PLoS ONE*.

[16] Limbourg A., Korff T., Napp L. C., Schaper W., Drexler H., Limbourg F. P. (2009). Evaluation of postnatal arteriogenesis and angiogenesis in a mouse model of hind-limb ischemia. *Nature Protocols*.

[17] Atala A., Kurtis Kasper F., Mikos A. G. (2012). Engineering complex tissues. *Science Translational Medicine*.

[18] Barbetta A., Bedini R., Pecci R., Dentini M. (2012). Role of X-ray microtomography in tissue engineering. *Annali dell'Istituto Superiore di Sanita*.

[19] Arkudas A., Beier J. P., Pryymachuk G. (2010). Automatic quantitative micro-computed tomography evaluation of angiogenesis in an axially vascularized tissue-engineered bone construct. *Tissue Engineering Part C: Methods*.

[20] Degani H., Chetrit-Dadiani M., Bogin L., Furman-Haran E. (2003). Magnetic resonance imaging of tumor vasculature. *Thrombosis and Haemostasis*.

[21] Xu H., Othman S. F., Magin R. L. (2008). Monitoring tissue engineering using magnetic resonance imaging. *Journal of Bioscience and Bioengineering*.

[22] Neeman M. (2002). Functional and molecular MR imaging of angiogenesis: seeing the target, seeing it work. *Journal of Cellular Biochemistry*.

[23] Boschi F., Marzola P., Sandri M. (2008). Tumor microvasculature observed using different contrast agents: a comparison between Gd-DTPA-Albumin and B-22956/1 in an experimental model of mammary carcinoma. *Magnetic Resonance Materials in Physics, Biology and Medicine*.

[24] Wang L. V., Hu S. (2012). Photoacoustic tomography: in vivo imaging from organelles to organs. *Science*.

[25] Hu S., Wang L. V. (2010). Photoacoustic imaging and characterization of the microvasculature. *Journal of Biomedical Optics*.

[26] Choi W. J., Wang R. K. (2014). In vivo imaging of functional microvasculature within tissue beds of oral and nasal cavities by swept-source optical coherence tomography with a forward/side-viewing probe. *Biomedical Optics Express*.

[27] Veksler B. A., Kuz'min V. L., Kobzev E. D., Meglinski I. V. (2012). The use of optical coherence tomography for morphological study of scaffolds. *Quantum Electronics*.

[28] Mahmud M. S., Cadotte D. W., Vuong B. (2013). Review of speckle and phase variance optical coherence tomography to visualize microvascular networks. *Journal of Biomedical Optics*.

[29] Srinivasan V. J., Radhakrishnan H. (2014). Optical coherence tomography angiography reveals laminar microvascular hemodynamics in the rat somatosensory cortex during activation. *NeuroImage*.

[30] Arribas S. M., Daly C. J., González M. C., Mcgrath J. C. (2007). Imaging the vascular wall using confocal microscopy. *Journal of Physiology*.

[31] Wang Z., Wei L., He Y., Li X., Shi G., Zhang Y. In vivo vascular imaging with adaptive optics confocal scanning fluorescence microscopy.

[32] Lewis J. D., Destito G., Zijlstra A. (2006). Viral nanoparticles as tools for intravital vascular imaging. *Nature Medicine*.

[33] Pittet M. J., Weissleder R. (2011). Intravital imaging. *Cell*.

[34] Motamedi S., Shilagard T., Edward K., Koong L., Qui S., Vargas G. (2011). Gold nanorods for intravital vascular imaging of preneoplastic oral mucosa. *Biomedical Optics Express*.

[35] van Zandvoort M., Engels W., Douma K. (2004). Two-photon microscopy for imaging of the (atherosclerotic) vascular wall: a proof of concept study. *Journal of Vascular Research*.

[36] Fu D., Matthews T. E., Ye T., Piletic I. R., Warren W. S. (2008). Label-free in vivo optical imaging of microvasculature and oxygenation level. *Journal of Biomedical Optics*.

[37] Genzel-Boroviczény O., Strötgen J., Harris A. G., Messmer K., Christ F. (2002). Orthogonal polarization spectral imaging (OPS): a novel method to measure the microcirculation in term and preterm infants transcutaneously. *Pediatric Research*.

[38] Son T., Lee J., Jung B. (2013). Contrast enhancement of laser speckle contrast image in deep vasculature by reduction of tissue scattering. *Journal of the Optical Society of Korea*.

[39] Mahé G., Humeau-Heurtier A., Durand S., Leftheriotis G., Abraham P. (2012). Assessment of skin microvascular function and dysfunction with laser speckle contrast imaging. *Circulation: Cardiovascular Imaging*.

[40] White S. M., Hingorani R., Arora R. P. S., Hughes C. C. W., George S. C., Choi B. (2012). Longitudinal in vivo imaging to assess blood flow and oxygenation in implantable engineered tissues. *Tissue Engineering—Part C: Methods*.

[41] Loghmani M. T., Warden S. J. (2013). Instrument-assisted cross fiber massage increases tissue perfusion and alters microvascular morphology in the vicinity of healing knee ligaments. *BMC Complementary and Alternative Medicine*.

[42] Wang X., Pang Y., Ku G., Xie X., Stoica G., Wang L. V. (2003). Noninvasive laser-induced photoacoustic tomography for structural and functional *in vivo* imaging of the brain. *Nature Biotechnology*.

[43] Cai X., Zhang Y. S., Xia Y., Wang L. V. (2013). Photoacoustic microscopy in tissue engineering. *Materials Today*.

[44] Xia J., Yao J., Wang L. V. (2014). Photoacoustic tomography: principles and advances. *Progress in Electromagnetics Research*.

[45] Kolkman R. G. M., Thumma K. K., Ten Brinke G. A. Photoacoustic imaging of tumor angiogenesis.

[46] Upputuri P. K., Wu Z., Gong L., Ong C. K., Wang H. (2014). Super-resolution coherent anti-Stokes Raman scattering microscopy with photonic nanojets. *Optics Express*.

[47] Upputuri P. K., Wen Z.-B., Wu Z., Pramanik M. (2014). Super-resolution photoacoustic microscopy using photonic nanojets: a simulation study. *Journal of Biomedical Optics*.

[48] Beard P. (2011). Biomedical photoacoustic imaging. *Interface Focus*.

[49] Lin R., Chen J., Wang H., Yan M., Zheng W., Song L. (2015). Longitudinal label-free optical-resolution photoacoustic microscopy of tumor angiogenesis in vivo. *Quantitative Imaging in Medicine and Surgery*.

[50] Oraevsky A. A., Wang L. V. Optical-resolution photoacoustic microscopy of angiogenesis in a transgenic mouse model.

[51] Talukdar Y., Avti P., Sun J., Sitharaman B. (2014). Multimodal ultrasound-photoacoustic imaging of tissue engineering scaffolds and blood oxygen saturation in and around the scaffolds. *Tissue Engineering—Part C: Methods*.

[52] Yun Nam S., Mallidi S., Zhang G., Suggs L. J., Emelianov S. Ultrasound and photoacoustic imaging to monitor vascular growth in tissue engineered constructs.

[53] Cai X., Zhang Y., Li L. (2013). Investigation of neovascularization in three-dimensional porous scaffolds *In Vivo* by a combination of multiscale photoacoustic microscopy and optical coherence tomography. *Tissue Engineering Part C: Methods*.

[54] Rudd J. H. F., Myers K. S., Bansilal S. (2008). Atherosclerosis inflammation imaging with 18F-FDG PET: carotid, iliac, and femoral uptake reproducibility, quantification methods, and recommendations. *Journal of Nuclear Medicine*.

